# Association between a marker of sperm DNA damage and sperm indices in infertile males in Benin City, Nigeria: A cross-sectional study

**DOI:** 10.18502/ijrm.v19i2.8472

**Published:** 2021-02-21

**Authors:** Ilyas Yusuf, Mathias Abiodun Emokpae

**Affiliations:** Department of Medical Laboratory Science, School of Basic Medical Sciences, College of Medical Sciences, University of Benin, Benin City, Nigeria.

**Keywords:** Male, Semen, Antioxidants, Sperm count, DNA damage.

## Abstract

**Background:**

Studies have shown oxidative DNA damage is associated with male infertility.

**Objective:**

This study determines the levels of 8-hydroxy-2'-deoxyguanosine (8-OHdG) and some markers of oxidative stress in seminal fluid of males investigated for infertility and men of proven fertility in Benin City, Nigeria.

**Materials and Methods:**

Semen samples produced by self or assisted masturbation were analyzed by microscopic technique according to the World Health Organization guidelines. Thereafter, samples were centrifuged and seminal fluid plasma separated and stored at -20°C prior to assay for 8-OHdG and oxidative stress biomarkers. Based on the sperm concentration/count, the overall samples were grouped into the following categories: normospermia (n = 20), oligozoospermia (n = 30), and azoospermia (n = 20). The control group comprised of 30 age-matched males of proven fertility. The seminal fluid 8-OHdG, total antioxidant status, superoxide dismutase and malondialdehyde (MDA) were assayed through ELISA and spectrophotometric methods, respectively.

**Results:**

Seminal plasma level of 8-OHdG and MDA were significantly higher (p = 0.01) in infertile subjects than controls. The mean levels of 8-OHdG and MDA in infertile subjects were higher in azoospermia than oligospermia than normospermia and so, was least in the normospermia. Conversely, the mean levels of total antioxidant status and superoxide dismutase were significantly lower (p = 0.01) in infertile than fertile the control male subjects with levels higher in normospermia than oligospermia and least in azoospermia. Moreover, the seminal 8-OHdG correlated negatively with sperm count (r = -0.359, p = 0.01), percent motility (r = -0.388, p = 0.04), and percent morphology (r = -0.327, p = 0.02).

**Conclusion:**

The assessment of sperm DNA damage in addition to routine seminal fluid analysis may play an important role in specific diagnosis and management of male infertility.

## 1. Introduction

Very few studies in Nigeria have investigated the role of sperm nuclear DNA integrity among infertile males. It has been suggested that sperm DNA integrity may be a potential predictor of male fertility and/or may serve as an adjunct to routine semen analysis. It has also been reported that some men who were unable to achieve pregnancy had sperm concentrations within normal range (1). Some have suggested that sperm of infertile men contain more DNA damage than fertile men and as such may have negative effects on fertility potential of these individuals (2, 3).

Male infertility is present in approximately half of all infertile couples. Currently, the routine analysis of male infertility includes aphysical examination and semen evaluation for sperm concentration, morphology, seminal volume, motility, and pH (4). Although, about 15% of patients with male factor infertility have been reported to have normal semen analysis (5), a conclusive and definitive diagnosis of male factor infertility can only be made through a routine semen analysis (4). Most laboratories in our setting currently rely on microscopic examination of semen in the diagnosis and management of the male infertility.

This baseline microscopic semen analysis may not sufficient to detect the cause (s) since the etiology of male infertility is multifactorial. The etiologies of male infertility could be anatomic defects, hormonal abnormalities, immune disorders, gene mutation, radiation, chemotherapy, ejaculatory failures, and environmental toxicants (6). In some cases, the identification of the exact cause (s) of male infertility may not be obvious and the established processes that led to the poor semen quality remain unclear (7). A standard semen analysis using a light microscope has been widely used in most laboratories for the initial assessment of male fertility potential; however, detecting defective sperm function by standard semen analysis is problematic because the spermatozoon is a highly specialized cell that exhibits manifold array of biological properties to achieve fertilization. In addition, results of standard semen analyses are selfhood or internal and subject to intra- and inter-observer variability (8). An individual's semen quality may differ due to several determinats such as days of abstinence from ejaculation, febrile illness, stress, and even problems with sample collection.

Oxidative stress is an important factor that can shape fertility potential, and high seminal reactive oxygen species (ROS) can cause sperm malfunction via lipid peroxidation of the sperm membrane. Therefore, low levels of ROS couple with low antioxidant defense can lead to redox imbalance, abnormal sperm motility, morphology, and sperm DNA damage. This is particularly because spermatozoa contain large amount of polyunsaturated fatty acids in their membranes. These make sperm cells to be susceptible to lipid peroxidation, loss of membrane integrity, reduced sperm motility, structural DNA damage, and apoptosis (9-14). The various spermatozoa abnormalities associated with infertility have been described (9, 15). There is a paucity of information on the association of oxidative stress with sperm DNA in infertile males in our setting. In addition, the lack of agreement on which infertile conditions should be evaluated for oxidative stress or which test should be conducted necessitated this study. The sperm DNA damage is an essential indicator of fertility potential often caused by oxidative stress, however, none of these is evaluated routinely in our setting. This study specifically focuses on investigating the extent of oxidative sperm DNA damage and to correlate sperm DNA damage with seminal fluid indices of men investigated for infertility.

## 2. Materials and Methods

### Study design

This cross-sectional study investigated 70 male subjects, aged 25-45 yr, for infertility between April and September, 2018. Of the 70 participants who met the inclusion criteria, 20 were normospermia, 30 oligozoospermia, and 20 azoospermia. They were included in the study because their partners were unable to conceive after one or more years of unprotected intercourse. On the other hand, the control group comprised of males without chronic clinical illnesses and those who had had their baby within the last one year.

### Inclusion criteria

All male subjects aged 25-45 years evaluated for infertility and consented to be enrolled without physical abnormalities or chronic illnesses were included in the study. Subjects without chronic clinical illnesses and those who had had their babies within the last one year and whose seminal fluid analysis showed over 15 million sperm cells/ml semen according to the World Health Organization (WHO) criteria were included and used as controls.

### Exclusion criteria

Individuals with known pathological or congenital conditions such as hypertension, diabetes mellitus, sexually transmitted diseases, testicular varicocele, and genital warts were excluded. In addition, individuals currently on antioxidant supplementation, smokers, alcoholics and those with known endocrine disease and physical abnormality were also excluded from the study.

### Sample collection

Semen samples were collected in a sterile container by self or assisted masturbation after at least 72 hr of sexual abstinence (without the use of spermicidal lubricants). The specimens were delivered to the laboratory within 30 min of ejaculation. Two specimens were collected at different visits within two months for analysis and mean value of the determinations was used. This is because spermatozoa are specialized cells that exhibit a diverse array of biological characteristics. The semen analyses results could be subjective and prone to intra and inter observer variability. Also, sperm quality of an individual can vary widely due to the duration of abstinence from coitus, febrile illness stress, and method of specimen collection. The sample size was calculated using sample size determination formula in health studies and the prevalence of 93% abnormal sperm parameters in all subjects visiting gynecological clinic (16).

### Sample preparation and laboratory analysis

#### Routine semen analysis

After liquefaction, the semen specimens were assessed for volume, appearance, pH, and viscosity. Routine semen analysis was performed microscopically with special interest in the sperm concentration, percent motility, and percent morphology. Based on the sperm concentration/count according to the WHO criteria (9), the overall samples were therefore grouped into the following categories: normospermia: ≥ 15 million sperm cells/ml semen; oligozoospermia: ≤ 15 million sperm cells /ml semen; and azoospermia: absence of sperm cells in the ejaculate. Thereafter, samples were centrifuged and the supernatant seminal fluid was separated into another clean and sterile plastic container. The seminal fluid plasma was then stored at -20°C prior to the assay of measured variables within two weeks.

Furthermore, the seminal fluid 8-hydroxy-2'-deoxy-goanosine (8-OHdG) was determined using enzyme-linked immunosorbent assay (ELISA) technique, while the total antioxidant status (TAS), superoxide dismutase (SOD), and malondialdehyde (MDA) were assayed by ELISA (Elabscience, Texas, USA) and spectrophotometric methods (North-West Life Science Specialties, Canada).

#### Assay of 8-OHdG (Elabscience, Texas, USA; catalog number: E; EL0028)

#### Principle

The ELISA kit uses the Comparative-ELISA principle. The microtiter plate is pre-coated with an antigen specific to 8-OHdG. During the reaction, the 8-OHdG in the sample or standard participates with a fixed amount of 8-OHdG on the solid phase supporter for sites on the biotinylated detection antibody specific to 8-OHdG. After a fixed period of reaction, horseradish peroxidase (HRP) and substrate solution are added and incubated. A color change develops and the absorbances are measured by the microplate reader. The concentration of the 8-OHdG in the sample is then determined by extrapolation from the standard curve.

#### Total antioxidant capacity (total antioxidant capacity T-AOC colorimetric assay kit [ABTS, enzyme method] Elabscience, Texas, USA; catalog number E-BC-K219-M)

#### Principle

The principle of the ABTS method for the assay of T-AOC is as follows. ABTS is oxidized to green ABTS${}^{+}$ by certain oxidant, which can be inhibited if there exist antioxidants. The T-AOC of the sample can be assayed and calculated by reading the absorbance of ABTS${}^{+}$ at 414 nm or 734 nm. Trolox is an analog of VE and has a similar antioxidant capacity as that of VE. Trolox is used as a reference for other antioxidants. Upon addition of the specimen, available antioxidant species present scavenge for ABTS${}^{+}$, after a certain reaction time, the amount of ABTS${}^{+}$ remaining is measured and expressed as Trolox equivalent.

#### SOD ELISA kit (Elabscience; catalog number E-EL_H 1113) 

#### Principle

The assay method is based on the Competitive ELISA principle. The micro ELISA plate provided in the kit is pre-coated with human SOD1. During the reaction, human SOD1 in the sample or standard competes with a fixed amount of human SOD1 on the solid phase supporter for sites on the biotinylated detection antibody specific to human SOD1. Surplus conjugate and unbound sample or standard are washed from the plate, and avidin conjugated to HRP are added to each microplate well and incubated. Then, a TMB substrate solution is included to each well. The enzyme-substrate reaction is stopped by the addition of stop solution and the color change is read spectrophotometrically at a wavelength of 450 nm ± 2 nm. The concentration of human SOD1 in the samples is then measured by comparing the absorbanceof the samples to the standard curve.

#### MDA assay kit (NWLSS, Canada)

#### Principle

This assay is based on the reaction, n of MDA with thiobarbituric acid (TBA), forming a MDA-TBA2 adduct that absorbs strongly at 532 nm.

### Ethical considerations

All included participants were informed about the nature of the study and informed consent was obtained prior to the specimens were collected. The study protocol was reviewed and approved by the Edo State Ministry of Health, Benin City (Reference number: HM.1208.355; dated: October 26, 2017).

### Statistical analysis

Data generated from the study were compared between the groups using the analysis of variance (ANOVA) by statistical software SPSS version 21 (SPSS Inc., Chicago, IL, USA). Post hoc comparison was done using unpaired Student's *t* test. Pearson's correlation coefficient was used to associate 8-OHdG with measured sperm indices. A p-value ≤ 0.05 was considered as statistically significant.

## 3. Results

No statistically significant differences were seen in age and semen volume between groups. The sperm concentration, motility and morphology percentages were significantly higher among normospermia than oligospermia (p = 0.01) (Table I). Table II shows the comparison of measured variables among studied participants and also indicates the post hoc multiple comparison of measured variables. The mean levels of 8-OHdG and MDA in normospermia, oligozoospermia, and azoospermia groups were significantly higher (p = 0.01) in the studied participants than the control group. The mean levels of 8-OHdG and MDA were higher among azoospermia than oligospermia and least in the normospermia. Conversely, the levels of TAS and SOD were significantly higher (p = 0.01) in the control group than the normospermia, oligozoospermia, and azoospermia infertile males. The mean levels of TAS and SOD were lowest among the azoospermia than the oligospermia and least in the normospermia. A significantly higher (p = 0.01) level of TAS was observed in the control group in comparison with the normospermia, oligozoospermia, and azoospermia groups. In the same vein, the mean value of SOD was significantly higher (p = 0.01) in the control group compared with the normospermia, oligozoospermia, and azoospermia infertile males. Seminal plasma 8-OHdG levels correlated negatively with sperm count (r = -035, p = 0.01), percent motility (r = -0388, p = 0.04), and percent morphology (r = -0.327, p = 0.02) (Table III).

**Table 1:** Comparison of measured sperm indices in studied participants


**Variables**	**Azoospermia (n = 20)**	**Oligospermia (n = 30)**	**Normospermia (n = 20)**	**Controls (n = 30)**	**P-value**
**Age (Yr)**	37.2 ± 0.8${}^{e}$	36.8 ± 1.1${}^{e}$	37.3 ± 0.9${}^{e}$	36.6 ± 0.9	0.91
**Concentration (10${}^{6}$/mL)**	0.00 ± 0.00	5.38 ± 0.40${}^{b}$	97.5 ± 14.0${}^{e}$	98.44 ± 13.89	0.01
**Total motility (%)**	0.00 ± 0.00	37.8 ± 4.6${}^{b}$	68.10 ± 3.50${}^{e}$	67.00 ± 3.90	0.01
**Morphology (%)**	0.00 ± 0.00	2.92 ± 0.03${}^{b}$	3.84 ± 0.01${}^{b}$	4.75 ± 0.02	0.01
**Volume (mL)**	3.00 ± 0.28${}^{e}$	3.16 ± 0.2${}^{e}$	3.41 ± 0.2${}^{e}$	3.4 ± 0.28	0.86
Data are expressed as Mean ± SD, (ANOVA) was used for multiple comparison of measured variables, ${}^{b}$P = 0.01, ${}^{e}$P > 0.05					

**Table 2:** Comparison of markers of DNA damage, oxidative stress, and lipid peroxidation


**Subjects**	**8 OHdG (ng/mL)**	**TAS (nmol/µL)**	**SOD (U/mL)**	**MDA (mmol/mL)**
**Controls (n = 30) A**	1.95 ± 0.47 (95%, 1.0, 2.9)	6.49 ± 0.28 (95%, 6.0, 7.0)	4.95 ± 1.46 (95%, 2.1, 6.9)	35.98 ± 4.12 (95%, 30.0, 43.4)
**Normospermia (n = 20) B**	3.58 ± 1.37${}^{a}$ (95%, 2.4, 7.5)	6.29 ± 0.23${}^{d}$ (95%, 6.0, 6.7)	4.74 ± 1.49${}^{e}$ (95%, 2.1, 6.8)	37.38 ± 3.50${}^{e,b}$ (95%, 31.4, 44.4)
**Oligospermia (n = 30) C**	4.31 ± 0.82${}^{a,e}$ (95% 1.5-10.0)	4.60 ± 0.25${}^{b,}$ (95% 4.1-5.0)	1.56 ± 0.24${}^{b}$ (95% 1.0-1.90)	43.4 ± 2.6${}^{b}$ (95% 36.1-48.6)
**Azoospermia (n = 20) D**	5.72 ± 2.69${}^{b,c}$ (95%, 2.0, 10.6)	4.45 ± 0.25${}^{b,c}$ (95%, 4.0, 4.8)	1.48 ± 0.24${}^{b,e}$ (95%, 1.0, 1.8)	45.91 ± 2.46${}^{b,a}$ (95%, 40.2, 48.7)
**P-value**	0.01	0.01	0.01	0.01
Data presented as Mean ± SD. Values in parentheses are confidence intervals. ANOVA was used to compare multiple measured variables${}^{a}$P = 0.03, ${}^{b}$P = 0.01, ${}^{c}$P = 0.02, ${}^{d}$P = 0.05, ${}^{e}$P > 0.05, 8 OHdG: 8-hydroxyguanosine, TAS: Total antioxidant status, SOD: Superoxide dismutase, MDA: Malondialdehyde				

**Table 3:** Correlation of 8-OHdG with measured sperm indices


**Measured semen parameters**		**Seminal fluid levels of 8-OHdG (ng/mL)**	**Correlation coefficient (R-value)**	**P-value**
**Sperm count (×10${}^{6}$ cells/mL)**				
	**>15**	3.54 ± 0.37	-0.360	0.01
	**≥5-14.9**	4.09 ± 0.71		
	**<5**	4.76 ± 0.40		
**Motility (%)**				
	**≥40%**	3.58 ± 1.37	-0.388	0.04
	**<40%**	4.76 ± 0.60		
**Morphology (%)**				
	**≥4%**	3.48 ± 0.62	0.327	0.02
	**<4%**	4.76 ± 0.81	
Data presented as Mean ± SD Pearson's correlation coefficient was used, 8-OHdG: 8-hydroxy-oxo-guanosine				

## 4. Discussion

The routine laboratory evaluation of males who failed to achieve conception naturally for one or more years includes at least two semen analyses for sperm count, percent motility, and percent morphology in order to establish the reproductive potential of the subjects. Assessment of markers of sperm DNA damage, oxidative stress, lipid peroxidation, and SOD activity are not routinely done even though their roles in male infertility are recognized (17-19).

The results from this study indicate that there is an evidence of oxidative DNA damage, higher lipid peroxidation, lower levels of measured antioxidant parameters among infertile males investigated for infertility, including among those whose sperm concentration was within normal reference range. The mean levels of 8-OHdG and MDA in infertile subjects increased from normospermia to oligospermia and azoospermia subjects. Conversely, the mean levels of TAS and SOD were significantly lower in infertile male than fertile control male subjects with levels decreasing from normospermic to oligosermic and azoospermic among infertile male subjects. Seminal 8-OHdG levels correlated negatively with sperm count, percent motility, and percent morphology in the study participants.

The observation of significantly higher 8-OHdG levels among infertile males than controls is consistent with previous studies (3, 20). Zini and colleagues reported that sperm DNA damage significantly correlated with abnormal sperm indices which were significantly higher in infertile males (25-27%) than their fertile counterparts (10-13%) (3). Similarly, it was observed that total sperm DNA damage (by Comet assay) correlated with sperm count, percent motility, and percent morphology among infertile males (20). Evgeni and co-authors also reported inverse correlation between the marker of DNA fragmentation and sperm indices in relation to fertility status in a Greek population (21). The possibility of achieving pregnancy naturally decreases with increasing levels of sperm DNA damage (22, 23). Among women undergoing in vitro fertilization (IVF) procedure, the rate of fertilization was reported to decrease from 58% to 38% (24). The evaluation of the extent of DNA damage in seminal plasma is imperative in order to predict fertilization rate and the risk of pregnancy outcome. Significantly higher sperm DNA damage was reported among oligoasthenoteratozoospermia than normozoospermia group (20).

Our group previously reported elevated levels of apoptotic markers among oligozoospermic infertile males in Zaria, Nigeria (24). The high apoptotic activity that takes place in seminal fluid of infertile males may help to explain the relationship of high sperm DNA damage with poor sperm indices.

The seminal plasma level of 8-OHdG increased with severity of sperm defects and count. Similar finding was reported which indicated that the incidence of abnormal morphologic defects has a significant relationship with abnormal chromatin structure and DNA strand breaks (25).

While the levels of TAS and SOD activity decreased with severity of measured sperm indices, the MDA levels increased with severity of defect in sperm parameters. It was reported that oxidative stress which causes sperm DNA damage also exacerbates lipid peroxidation of sperm plasma membrane. This may ultimately lead to structural and functional damage in the sperm cells (26). Increased oxidative stress can negatively affect sperm motility by damaging the axonemal architecture and/or reducing the intra-cellular adenosine triphosphate levels.

Sperm DNA damage that has been correlated with high levels of ROS play an important role in male factor infertility and adversed reproductive outcomes (27). At low levels, ROS play an important role in sperm growth, development and functions such as capacitation and the acrosome reaction. Seminal plasma contains antioxidants that help to safeguard sperm DNA. However, when an elevated amount of ROS is generated beyond the antioxidant capacity, sperm DNA damage may occur (28). It has been suggested that sperm DNA integrity may be a potential predictor of male fertility than routine semen analysis. This finding has supported the fact that the sperm of infertile men contain more DNA damage than fertile men and that this sperm DNA damage may have a negative effect on fertility potential of such subjects (2, 3). The central role of oxidative stress in the etiology of sperm DNA damage cannot be overemphasized. These findings further point to the harmful role of oxidative stress in the etiology of male infertility.

The origin of ROS in semen includes leukocytes and the sperm themselves, particularly immature sperm with cytoplasmic retention and abnormal head morphology distinguished by retention of residual cytoplasm (29). Both leukocytospermia and retention of residual cytoplasm within sperm have been linked with increased sperm DNA damage probably secondary to increased ROS level produced by these cells (30).

Similarly, the significantly higher levels of MDA observed among the oligospermia and azoospermia than controls may further point to higher levels of lipid peroxidation among these groups of infertile males. ROS bombard all major classes of biological molecules, mainly the polyunsaturated fatty acids (PUFA) of cell membranes. The oxidative damage of PUFA, known as lipid peroxidation, is particularly destructive, because it proceeds as a “self-perpetuating chain reaction” (31).

The significantly higher levels of TAS and SOD in control group imply an optimal antioxidants activity resulting in scavenging of sperm-specific ROS and ultimately protecting the spermatozoa from oxidative alterations. Mature spermatozoa are susceptible to oxidative attacks because the membranes are rich in PUFA that are easily oxidized by ROS. When oxidized in the presence of excess ROS, these PUFA would amplify the generation of ROS in a vicious oxidative stress circle leading to both cellular and DNA oxidative alterations (32, 33). In addition, superoxide dismutase known to be the most important antioxidant enzyme and a strong scavenger of ROS has been reported to be produced by the Leydig and Sertoli cells in the testis (34).

## 5. Conclusion

Seminal plasma levels of 8-OHdG and MDA were significantly higher in infertile subjects than controls. The mean levels of 8-OHdG and MDA in infertile subjects increased with increased abnormality of measured sperm parameters from normospermia to oligozoospermia and azoospermia. On the other hand, the mean levels of TAS and SOD were significantly lower in infertile male than fertile control male subjects with levels decreasing with increased abnormality of sperm indices. Seminal plasma 8-OHdG correlated negatively with sperm count, percent motility, and percent morphology. The assessment of sperm DNA damage in addition to routine seminal fluid analysis may play an important role in specific diagnosis and management of male infertility.

##  Conflict of Interest

The authors declare no conflict of interest.

## References

[1] Gharreb DA, Sarhan EME. Role of oxidative stress in male fertility and Idiopathic infertility: Causes and treatment. *J Diagn Tech Biomed Anal* 2014; 3: 1000110.

[2] Guzick DS, Overstreet JW, Factor-Litvak P, Brazil CK, Nakajima ST, Coutifaris C. Sperm morphology, motility, and concentration in fertile and infertile men. *N Engl J Med* 2001; 345: 1388–1393. 10.1056/NEJMoa003005 11794171

[3] Zini A, Bielecki R, Phang D, Zenzes MT. Correlations between two markers of sperm DNA integrity, DNA denaturation and DNA fragmentation, in fertile and infertile men. *Ferti Steril* 2001; 75: 674–677. 10.1016/s0015-0282(00)01796-9 11287017

[4] Schulte RT, Ohl DA, Sigman M, Smith GD. Sperm DNA damage in male infertility: Etiologies, assays and outcomes. *J Assist Reprod Genet *2010; 27: 3–12. 10.1007/s10815-009-9359-x PMC2826626 20012685

[5] Agarwal A, Allamaneni ShSR. Sperm DNA damage assessment: A test whose time has come. *Fertil Steril* 2005; 84: 850–853. 10.1016/j.fertnstert.2005.03.080 16213833

[6] Uadia PO, Emokpae AM. Male infertility in Nigeria: A neglected reproductive health issue requiring attention. *J Basic Clin Reprod Sci* 2015; 4: 45–53.

[7] Emokpae MA, Uadia PO. Potential risk of senescence on male fertility and sperm DNA damage on progeny. *Trop J Nat Prod Res* 2017; 1: 58–62.

[8] Jungwirth A, Diemer T, Dohle GR, Giwercman A, Kopa Z, Krausz C, et al. Guidelines on male infertility. *Eur Urol* 2015; 62: 324–332. 10.1016/j.eururo.2012.04.048 22591628

[9] World Health Organization. WHO laboratory manual for the examination and processing of human semen. 5${}^{\mathrm{th}}$ Ed. Geneva: World Health Organization; 2010.

[10] Ni K, Steger K, Yang H, Wang H, Hu K, Zhang T, et al. A comprehensive investigation of sperm DNA damage and oxidative stress injury in infertile patients with subclinical, normozoospermic, and astheno/oligozoospermic clinical varicocoele. *Andrology* 2016; 4: 816–824. 10.1111/andr.12210 27218783

[11] Sabeti P, Pourmasumi S, Rahiminia T, Akyash F, Talebi AR. Etiologies of sperm oxidative stress. *Int J Reprod Biomed* 2016; 14: 231–240. PMC4918773 27351024

[12] Bisht Sh, Faiq M, Tolahunase M, Dada R. Oxidative stress and male infertility. *Nat Rev Urol* 2017; 14: 470–485. 10.1038/nrurol.2017.69 28508879

[13] Wagner H, Cheng JW, Ko EY. Role of reactive oxygen species in male infertility: An updated review of literature. *Arab J Urol* 2017; 16: 35–43. 10.1016/j.aju.2017.11.001 PMC5922220 29713534

[14] Alahmar AT. Role of oxidative stress in male infertility: An update review. *J Hum Reprod Sci* 2019; 12: 4–18. 10.4103/jhrs.JHRS_150_18 PMC6472207 31007461

[15] Cooper TG, Noonan E, von Eckardstein S, Auger J, Baker HWG, Behre HM, et al. World Health Organization reference values for human semen characteristics. *Hum Reprod Update* 2010; 16: 231–245. 10.1093/humupd/dmp048 19934213

[16] Chukudebelu WO. The male factor in infertility - Nigerian experience. *Int J Fertil* 1978; 23: 238–239. 40901

[17] Uadia PO, Emokpae MA. Implications of oxidative stress on male infertility. *Trans Niger Soc Biochem Mol Biol* 2015; 1: 19–29.

[18] Emokpae MA, Chima HN, Ahmed M. Seminal plasma caspase 3, cytochrome c and total antioxidant capacity in oligospermic males and association with sperm indices. *J Exp Integr Med* 2016; 6: 1–4.

[19] Emokpae MA, Uadia PO, Omole-Ohonsi A. Pattern of hormonal abnormalities and association with sperm parameters among oligospermic male partners of infertile couples. *Niger Endocrin Pract* 2014; 8: 13–19.

[20] Aydos OS, Yukselten Y, Kaplan F, Sunguroglu A, Aydos K. Analysis of the correlation between sperm DNA integrity and conventional semen parameters in infertile men. *Turk J Urol* 2015; 41: 191–197. 10.5152/tud.2015.98475 PMC4621147 26623148

[21] Evgeni E, Lymberopoulos G, Gazouli M, Asimakopoulos B. Conventional semen parameters and DNA fragmentation in relation to fertility status in a Greek population. *Eur J Obstet Gynecol Reprod Biol* 2015; 188: 17–23. 10.1016/j.ejogrb.2015.02.026 25770843

[22] Majzoub A, Arafa M, Mahdi M, Agarwal A, Al Said S, Al-Emadi I, et al. Oxidation-reduction potential and sperm DNA fragmentation, and their associations with sperm morphological anomalies amongst fertile and infertile men. *Arab J Urol* 2018; 16: 87–95. 10.1016/j.aju.2017.11.014 PMC5922185 29713539

[23] Host E, Lindenberg S, Smidt-Jensen S. The role of DNA strand breaks in human spermatozoa used for IVF and ICSI. *Acta Obstet Gynecol Scand* 2000; 79: 559–563. 10929955

[24] Emokpae MA, Chima HN, Ahmed M. Seminal Caspase 3, cytochrome c and total antioxidant capacity in Nigerian male subjects undergoing infertility evaluation. *Afr J Biomed Res *2019; 22: 59–63.

[25] Ombelet W, Wouters E, Boels L, Cox A, Janssen M, Spiessens C, et al. Sperm morphology assessment: Diagnostic potential and comparative analysis of strict or WHO criteria in a fertile and a subfertile population. *Int J Androl* 1997; 20: 367–372. 10.1046/j.1365-2605.1998.00079.x 9568530

[26] Agarwal A, Said TM. Oxidative stress, DNA damage and apoptosis in male infertility: A clinical approach. *BJU Int* 2005; 95: 503–507. 10.1111/j.1464-410X.2005.05328.x 15705068

[27] Barroso G, Morshedi M, Oehninger S. Analysis of DNA fragmentation, plasma membrane translocation of phosphatidylserine and oxidative stress in human spermatozoa. *Hum Reprod* 2000; 15: 1338–1344. 10.1093/humrep/15.6.1338 10831565

[28] Aitken RJ, Krausz C. Oxidative stress, DNA damage and the Y chromosome.* Reproduction* 2001; 122: 497–506. 10.1530/rep.0.1220497 11570956

[29] Ollero M, Gil-Guzman E, Lopez MC, Sharma RK, Agarwal A, Larson K, et al. Characterization of subsets of human spermatozoa at different stages of maturation: Implications in the diagnosis and treatment of male infertility. *Hum Reprod *2001; 16: 1912–1921. 10.1093/humrep/16.9.1912 11527898

[30] Fischer MA, Willis J, Zini A. Human sperm DNA integrity: Correlation with sperm cytoplasmic droplets. *Urology* 2003; 61: 207–211. 10.1016/s0090-4295(02)02098-8 12559297

[31] Aitken RJ, Curry BJ. Redox regulation of human sperm function: From the physiological control of sperm capacitation to the etiology of infertility and DNA damage in the germ line. *Antioxid Redox Signal* 2011; 14: 367–381. 10.1089/ars.2010.3186 20522002

[32] Burruel V, Klooster KL, Chitwood J, Ross PJ, Meyers SA. Oxidative damage to rhesus macaque spermatozoa results in mitotic arrest and transcript abundance changes in early embryos. *Biol Reprod* 2013; 89: 72. 10.1095/biolreprod.113.110981 PMC4094196 23904511

[33] Moazamian R, Polhemus A, Connaughton H, Fraser B, Whiting S, Gharagozloo P, et al. Oxidative stress and human spermatozoa: Diagnostic and functional significance of aldehydes generated as a result of lipid peroxidation. *Mole Hum Reprod* 2015; 21: 502–515. 10.1093/molehr/gav014 25837702

[34] Mruk DD, Cheng CY. In vitro regulation of extracellular superoxide dismutase in sertoli cells. *Life Sci *2000; 67: 133–145. 10.1016/s0024-3205(00)00609-3 10901281

